# GCI: a continuity inspector for complete genome assembly

**DOI:** 10.1093/bioinformatics/btae633

**Published:** 2024-10-21

**Authors:** Quanyu Chen, Chentao Yang, Guojie Zhang, Dongya Wu

**Affiliations:** International Institutes of Medicine, The Fourth Affiliated Hospital, Zhejiang University School of Medicine, Yiwu 322000, China; Center for Evolutionary & Organismal Biology, Liangzhu Laboratory, Zhejiang University Medical Center, Hangzhou 311121, China; Chu Kochen Honors College, Zhejiang University, Hangzhou 310058, China; BGI Research, Shenzhen 518083, China; BGI Research, Wuhan 430074, China; International Institutes of Medicine, The Fourth Affiliated Hospital, Zhejiang University School of Medicine, Yiwu 322000, China; Center for Evolutionary & Organismal Biology, Liangzhu Laboratory, Zhejiang University Medical Center, Hangzhou 311121, China; Women’s Hospital, School of Medicine, Zhejiang University, Hangzhou 310006, China; Center for Evolutionary & Organismal Biology, Liangzhu Laboratory, Zhejiang University Medical Center, Hangzhou 311121, China

## Abstract

**Motivation:**

Recent advances in long-read sequencing technologies have significantly facilitated the production of high-quality genome assembly. The telomere-to-telomere (T2T) gapless assembly has become the new golden standard of genome assembly efforts. Several recent efforts have claimed to produce T2T-level reference genomes. However, a universal standard is still missing to qualify a genome assembly to be at T2T standard. Traditional genome assembly assessment metrics (N50 and its derivatives) have no capacity in differentiating between nearly T2T assembly and the truly T2T assembly in continuity either globally or locally. Additionally, these metrics are independent of raw reads, making them inflated easily by artificial operations. Therefore, a gaplessness evaluation tool at single-nucleotide resolution to reflect true completeness is urgently needed in the era of complete genomes.

**Results:**

Here, we present a tool called Genome Continuity Inspector (GCI), designed to assess genome assembly continuity at single-base resolution, and evaluate how close an assembly is to the T2T level. GCI utilizes multiple aligners to map long reads from various sequencing platforms back to the assembly. By incorporating curated mapping coverage of high-confidence read alignments, GCI identifies potential assembly issues. Meanwhile, it provides GCI scores that quantify overall assembly continuity on the whole genome or chromosome scales.

**Availability and implementation:**

The open-source GCI code is freely available on Github (https://github.com/yeeus/GCI) under the MIT license.

## 1 Introduction

Long-read sequencing technologies, such as PacBio High-Fidelity (HiFi) and Oxford Nanopore Technology (ONT), are now routinely employed in de novo genome assembling pipelines. These technologies have demonstrated their capability to address assembly challenges in highly repetitive regions, as seen in several gapless genome assemblies, including human (Nurk *et al.* 2022, Yang *et al.* 2023), chicken (Huang *et al.* 2023), *Arabidopsis thaliana* (Naish *et al.* 2021), and rice (Song *et al.* 2021). A series of metrics are currently used to evaluate the quality of de novo genome assemblies based on the “3C criterion” (completeness, correctness and continuity) (Wang and Wang 2023). Completeness, is often assessed using Benchmarking Universal Single-Copy Orthologs (BUSCO), Core Eukaryotic Genes Mapping Approach (CEGMA), and similar gene-mapping based tools (e.g. asmgene) (Li 2018), but these gene-focus assessments may not accurately represent the quality of gene-desert regions with complex structures. *K*-mer completeness evaluated by Merqury provides another completeness indicator, but is sensitive to low read quality and experimental contamination, potentially introducing false or exogenous *K*-mers (Rhie *et al.* 2020). For correctness, consensus quality value (QV) is widely used to measure shared *K*-mers between raw reads and the final assembly, although it can be artificially manipulated by removing erroneous assembly sequences (Rhie *et al.* 2020).

Genome assembly continuity is typically measured by the metric contig N50, which denotes the length of the shortest contig at which the total length of all contigs of that size or longer equals half of the total assembly sequence length. However, contig N50 and its derivative NG50, auN (https://lh3.github.io/2020/04/08/a-new-metric-on-assembly-contiguity) or E-size values (Salzberg *et al.* 2012), have easily reached or approaching their theoretical maximums due to the nature of contig N50's discontinuity. This limitation suggests that these metrics have a reduced capacity to differentiate between assemblies from different individuals using long reads or to reflect improvements in assembly quality. In other words, once the contig N50 reaches the value of chromosome N50 length, further improvements in gap filling may not be captured by the contig N50 metric. Furthermore, assembly continuity could be artificially inflated by directly replacing or removing gaps, which cannot be detected solely from the assembly sequences. Therefore, there is an urgent need for tools that can detect assembly errors at base-level resolution by realigning raw reads to ensure the authenticity of a truly gapless assembly.

Mapping long reads back against genome assembly can reveal abnormal signals (e.g. mapping quality, clipping information, read coverage, and edit distance/mismatch), which could be used to identify potential assembly errors. To detect base-resolution assembly errors, several tools have been developed using this strategy, including Flagger (Liao *et al.* 2023) and CRAQ (Li *et al.* 2023). The T2T-polish pipeline developed by human genome T2T consortium (hereafter called as T2T-polish) also includes a sub-module designed for this purpose (Mc Cartney *et al.* 2022). Flagger, developed by Human Pangenome Reference Consortium (HPRC), has been applied in the human pangenome study (Liao *et al.* 2023). It detects anomalies in read coverage and partitions the assembly into different categories predicting the accuracy of the assembly, such as duplicated, collapsed, and erroneous blocks. The T2T-polish pipeline similarly reports the assembly issue regions primarily based on read mapping coverage.Clipping information for Revealing Assembly Quality (CRAQ) focuses on clipping information from read alignments to detect potential assembly errors, but ignores the regions with extremely low or high coverage (Li *et al.* 2023). Briefly, abnormal mapping signals are collected as assembly issues based on reads coverage for Flagger and T2T-polish, and clipping information for CRAQ. While such approaches are effective, it is important to note that sequencing bias in different genomic regions and aligning bias in highly repetitive regions by different aligners can also produce mapping anomalies, potentially leading to false positives in the detection of assembly issues.

Here, we present a new alignment-based evaluator called Genome Continuity Inspector (GCI) for assessing genome assembly quality, particularly targeting assembles at or near T2T level. GCI integrates alignments of long reads from multiple sequencing platforms back to the assembly and multiple aligners. Instead of detecting issues using abnormal read mapping signals, GCI calls potential assembly issues based on curated coverage of high-confidence read alignments. Additionally, GCI calculates scores to quantify the overall continuity of a genome assembly at the genome or chromosome levels. In summary, GCI provides a new strategy to evaluate the quality of genome assembly, particularly in the T2T era.

## 2 Materials and methods

### 2.1 Overview of GCI

#### 2.1.1 Reads mapping and filtering

**Figure 1.**
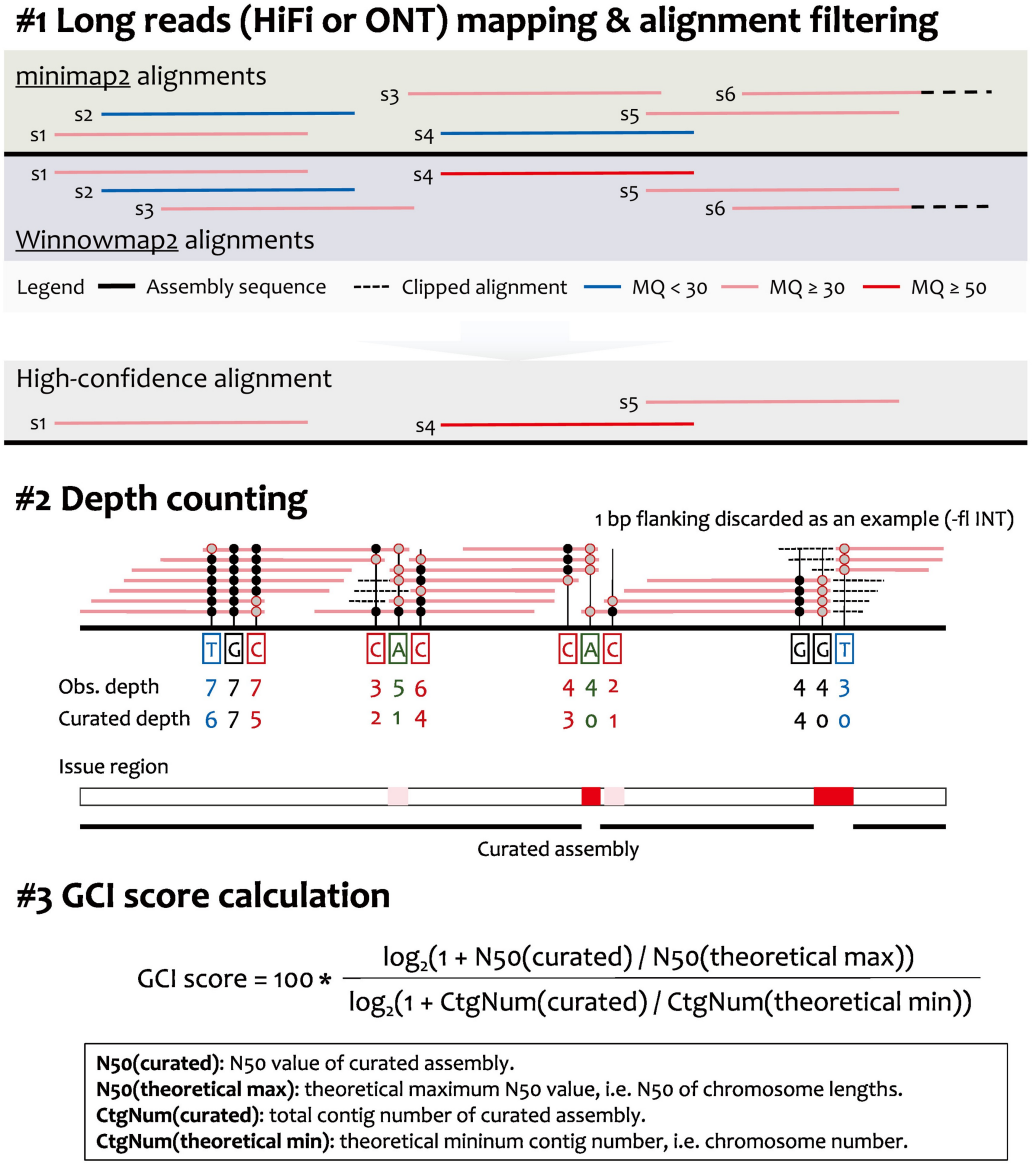
Workflow of GCI. Multiple alignment strategies (e.g. minimap2 and Winnowmap2) are used in mapping HiFi or ONT reads against the assembly sequence, resulting in two alignment outcomes for each read. Following a series of stringent integration and filtering steps, high-confidence alignments are curated and kept. By trimming both ends of the read alignment, the curated depth is counted for each reference base. Potential assembly issues are identified based on zero or extremely low depth. A curated assembly is produced by replacing assembly issues with gaps. To profile the overall genome-wide continuity of assembly, GCI scores are calculated by considering both the contig N50 values and contig numbers of the curated assembly and theoretically gapless assembly. Obs., observed; CtgNum, contig number.

GCI is a computational pipeline that uses alignment files (in BAM or PAF format), generated by mapping long reads (HiFi and ONT reads) back to the final assembly, as inputs. It outputs a score as an indicator of assembly continuity and reports potential assembly issues. The tool requires alignments that pass stringent filtering criteria. All unmapped, secondary and supplementary alignments are discarded. Moreover, mapping quality (<30 in default), mapping identity (<90% in default) and clipped proportion (>10% in default) are further employed to remove low-quality alignments. To address potential alignment biases introduced by different mapping algorithms among aligners, GCI recommends using at least two popular sequence aligners [e.g. minimap2 (Li 2018), Winnowmap2 (Jain *et al.* 2022), VerityMap (Bzikadze *et al.* 2022)] on the same dataset (Fig. 1). Alignments from the two aligners that meet the mapping quality requirements and have consistent mapping coordinates (with overlap ≧90% by default) are kept. The mapping accuracy and sensitivity are prominently different between aligners. For example, minimap2 runs much faster than Winnowmap2 and VerityMap but underperforms in aligning highly repetitive sequences, with low mapping quality (usually approaching to zero) (Bzikadze *et al.* 2022, Jain *et al.* 2022). Therefore, to rescue alignments in repetitive regions, read alignments are kept if one aligner produces high mapping quality (≧50 by default) for those reads.

#### 2.1.2 Depth counting

After a series of strict alignment filtration, we count the mapping coverage for each base. Instead of directly using samtools depth for observed depth, several bases at both ends of an alignment are firstly trimmed and not used for depth counting (one base is shown as an example in Fig. 1, with the trimming length being user-defined). This approach aims to exclude potentially clipped alignments and enhance the sensitivity in detecting potential assembly gaps with insufficient read mapping support.

#### 2.1.3 GCI score calculation

According to the curated mapping depth for each base, GCI reports the potential assembly issues, where the regions have zero or extremely low depth. Physically adjacent issues (e.g. distance less than 0.5% of chromosome length) are merged. The original chromosomes or sequences are subsequently split into curated contigs at loci with no sufficient read alignment supporting. The curated contig N50 and number are calculated for the curated assembly. Finally, considering the discontinuity of contig N50, GCI integrates both the contig N50 value and the contig number of the curated assembly to quantify the gap of the assembly and a truly gapless T2T assembly, using a GCI score (scaled from zero to 100) (Fig. 1). Even if the contig N50 value has been saturated, the contig numbers could be used to quantify the continuity differences between assemblies. For a true T2T assembly, no issues or gaps would be detected and thus the curated contig N50 equals to the theoretical maximum (chromosome N50) and the contig number equals to the number of chromosomes, which will thus produce a GCI score of 100.

#### 2.1.4 Output

Potential assembly issue regions with zero or low-depth read alignment support, and GCI scores for whole-genome assembly and each chromosome are reported. Additionally, curated mapping depth plots are available for manual check.

### 2.2 Datasets

Several high-quality genomes have been released recently and some claimed to be at or near the T2T level, including several human genomes [CHM13 (Nurk *et al.* 2022), CN1 (Yang *et al.* 2023), and HG002 (Jarvis *et al.* 2022)], and other model organisms {chicken [GGswu (Huang *et al.* 2023)], *Arabidopsis* (Col-CEN v1.2, Naish *et al.* 2021) and rice (MH63) (Song *et al.* 2021)}. To demonstrate the performance of GCI workflow in assessing quality of genome assembly, we downloaded the genome assemblies and corresponding raw long reads (HiFi and ONT) for these genomes and performed the assessment with GCI. All HiFi reads were firstly filtered using HiFiAdapter (Sim *et al.* 2022). For a haploid (i.e. CHM13), highly homozygous or self-fertilized (i.e. Col-CEN and MH63) or unphased (i.e. GGswu) diploid assembly, long reads were mapped directly against the corresponding assembly. For haplotype-resolved diploid assemblies (i.e. human genomes CN1 and HG002), ONT and HiFi reads were firstly phased into paternal and maternal haplotypes based on parental genomic information using Canu (Nurk *et al.* 2020). The unphased HiFi and ONT reads were randomly and averagely assigned to the two haplotypes. Due to the lack of chromosome Y in CHM13, chromosome Y was not evaluated for the three human genomes. Plastid (mitochondria and chloroplast) genomes were excluded before aligning. The computational resources consumed in evaluating the human (CHM13), *Arabidopsis* (Col-CEN), and rice (MH63) genome assemblies using the whole GCI workflow were documented in Supplementary Table S1.

### 2.3 Comparison among assembly issue detection tools

Two base-resolution quality evaluators, CRAQ (https://github.com/JiaoLaboratory/CRAQ) and T2T-polish (https://github.com/arangrhie/T2T-Polish), were used to detect potential assembly issues for the genomes analyzed in this study and were compared against GCI’s performance. For GCI, alignment BAM or PAF files generated by minimap2 and Winnowmap2 using all available long reads (HiFi and ONT) were input. CRAQ requires a single long read alignment as input, thus we provided the ONT read alignments produced by Winnowmap2, due to the superior continuity of ONT reads. As recommended by CRAQ, NGS read alignments were also supplied. For the T2T-polish pipeline, HiFi and ONT read alignments were input separately and the resulting issue regions were integrated to produce a final dataset of assembly issues. Default parameters were used in both CRAQ and T2T-polish pipeline.

Additionally, we assessed the performance of GCI, CRAQ, and T2T-polish using simulated datasets. We introduced varying numbers of simulated issues (10, 20, and 40) into the high-confidence genomic regions of the *Arabidopsis* genome (Col-CEN v1.2). Regions where none of the three tools (GCI, CRAQ, and T2T-polish) detected issues were considered as high-confidence regions. The issue loci detected by any one of the three tools in the real assembly and their 100-Kb flanking regions were excluded from the simulation. Insertions (INSs) with the lengths from 10 to 50 Kb and deletions (DELs) with the lengths from 50 to 100 Kb were artificially introduced in the assembly, respectively. The length range of the simulated issues was considered according to the sequencing length range of HiFi and ONT reads. The insertion sequences were randomly copied from other regions across the genome. Using the same raw reads, each tools (GCI, CRAQ, and T2T-polish) was used to detect issues in each simulation run. Precision, recall, and F1 score were calculated, with five replicates performed for each simulated issue size (5 INSs + 5 DELs, 10 INSs + 10 DELs, 20 INSs+ 20 DELs). We also separately evaluated the performance in simple versus highly repetitive complex regions (including centromere and rDNA regions).

## 3 Results

### 3.1 GCI score shows higher sensitivity in evaluating assembly continuity for high-quality genomes

**Figure 2.**
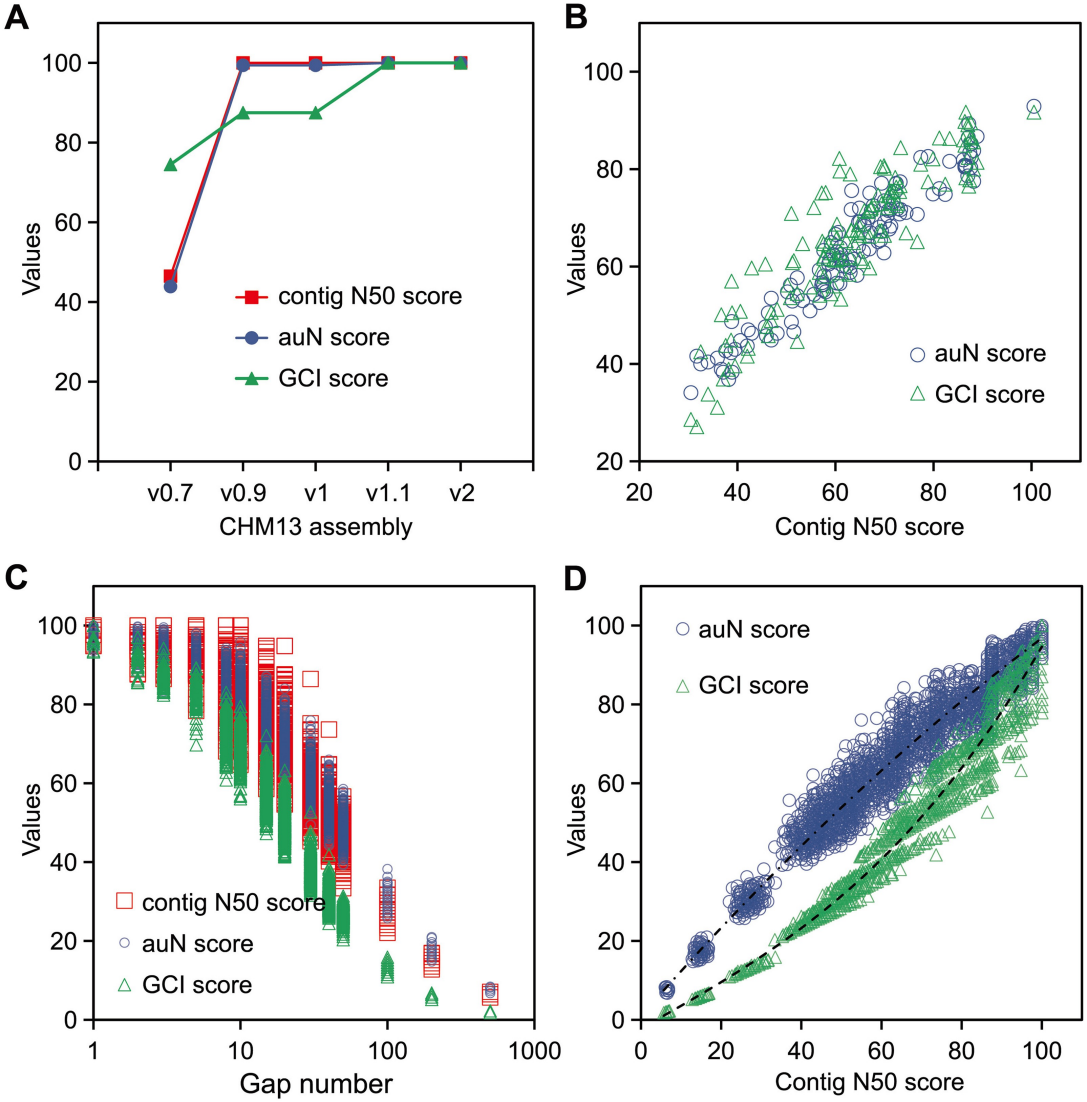
Sensitivity assessment of contig N50, auN, and GCI score in quantifying assembly continuity. (A) Contig N50, auN, and GCI scores for various versions of the CHM13 assembly. Contig N50 and auN were standardized from zero to 100 based on their theoretical maximum values, respectively. (B) auN and GCI scores for human phased genome assemblies from the HGSVC phase 3. (C) Simulation of different gap numbers (1, 2, 3, 5, 8, 10, 15, 20, 30, 40, 50, 100, 200, and 500) in a human haploid genome, with 200 times of simulation for each gap size. (D) Simulated curves of auN and GCI scores with different contig N50 scores.

We evaluated the sensitivity of assessing assembly continuity using GCI score, contig N50 and its derived metric auN with both publicly released genomes and simulated data. To make them comparable, we firstly scaled contig N50 and auN values as scores ranging from zero to 100 based on their theoretical maximums, respectively. CHM13 was the first human genome to be completely assembled, with multiple versions of updates. Most gaps (84/89) in v0.7 were filled in v0.9 and the remaining five rDNA gaps were resolved in v1.1 (https://github.com/marbl/CHM13). Contig N50 and auN had achieved their theoretical maximums since v0.9 and the continuity improvement from filling rDNA gaps was not reflected by them, while GCI score effectively captured this change (Fig. 2A). Additionally, we calculated these three metrics for population-level human phased genome assemblies from the Human Genome Structural Variation Consortium (HGSVC) phase 3. Despite similar contig N50 scores, GCI scores showed greater deviations than those of auN, suggesting GCI’s superior capacity in distinguishing continuity (Fig. 2B). By randomly simulating gaps in the genome of CHM13, we found that contig N50 was the least sensitive while GCI scores exhibited highest rate of decline with increasing gap numbers, particularly for nearly complete assemblies with few gaps (Fig. 2C). Moreover, a steeper slope was observed for GCI compared to auN as contig N50 approached its maximum (Fig. 2D). This indicates, GCI scores are more effective in highlighting and distinguishing continuity for high-quality assemblies, whether for reflecting assembly improvement or comparing inter-individual quality.

### 3.2 GCI evaluation for human genome assemblies

We evaluated GCI performance using three state-of-the-art human genome assemblies (CHM13, CN1, and HG002). CHM13 and CN1 assemblies were gapless, whose contig N50 and auN values were the theoretical maximum, while HG002 phased assemblies were fragmented with lower contig N50 and auN values (Table 1). GCI evaluation based on ONT and HiFi reads varied, where GCI scores based on ONT reads were more than twice higher than those using HiFi reads for all three human genomes. This highlights the crucial role of ONT reads in enhancing assembly continuity, in spite of their relatively lower base accuracy. Therefore, we recommended using both HiFi and ONT reads for GCI evaluation. When assessed with both HiFi and ONT reads, the curated N50 value for the CHM13 assembly reached its theoretical maximum N50 value, while the values for CN1 and HG002 were lower than the observed contig N50 values calculated from raw assemblies, indicating fewer assembly issues in the CHM13 assembly (Table 1). Consistently, the haploid CHM13 outperformed the two haplotype-resolved diploid human genomes, achieving a GCI score of 87.04, compared to 66.79 (maternal) and 77.90 (paternal) for CN1, and 18.72 (maternal) and 27.78 (paternal) for HG002. This was expected since fewer issues were reported in CHM13 assembly due to its higher homozygosity.

**Table 1.** GCI evaluation for the genome assemblies of model species.

Species	Human					Chicken	*Arabidopsis*	Rice
Assembly	CHM13 (v.2.0)	CN1.mat (v.0.9)	CN1.pat (v.0.9)	HG002.mat.cur.20211005	HG002.pat.cur.20211005	GGswu	Col-CEN (v1.2)	MH63 (RS3)
Genome size (Mb)	3055	3035	2875	3001	2852	1101	132	396
Theoretical maximum contig N50 (Mb)	154.26	157.37	145.80	154.41	146.75	91.36	25.74	31.92
Contig N50 (Mb)	154.26	157.37	145.80	62.88	84.93	91.36	25.74	31.92
E-size/auN (Mb)	156.45	156.10	156.39	73.54	77.20	93.34	26.98	33.99
HiFi depth (×)	∼58	∼44	∼44	∼83	∼83	∼51	∼90	∼39
Curated contig N50 (Mb) (HiFi)	102.83	73.85	66.46	29.22	40.18	19.25	14.28	24.81
GCI (HiFi)	41.83	22.84	22.47	7.26	11.94	7.99	30.75	49.89
ONT depth (×)	∼134	∼39	∼39	∼257	∼257	∼103	∼560	NA
≥ 100 kb (ultra-long) ONT depth (×)	∼39	∼20	∼20	∼34	∼34	∼10	∼4	NA
Curated contig N50 (Mb) (ONT)	154.26	132.02	111.00	58.82	81.56	73.41	25.74	NA
GCI (ONT)	87.04	51.54	63.04	18.39	27.16	30.42	99.99	NA
Curated contig N50 (Mb) (HiFi + ONT)	154.26	137.88	134.86	58.82	81.56	73.41	25.74	NA
GCI (HiFi+ONT)	87.04	66.79	77.90	18.72	27.78	29.37	99.99	NA

Differential GCI scores and high-confidence read mapping supports were observed between the two haplotypes in diploid genomes, highlighting the heterogeneity in assembly difficulty due to potentially haplotype-specific complex sequences (Table 1; Supplementary Fig. S1). While sequencing depth of long reads, especially for ultra-long ONT reads (≧100 kb), is crucial for improving the continuity, we noticed that HG002, assembled with a higher quantity of HiFi and ONT reads, exhibited lower GCI scores compared to CN1 (Table 1). This discrepancy suggests that differential assembly algorithms and gap-filling strategies might contribute to the observed difference in continuity between CN1 and HG002.

**Figure 3.**
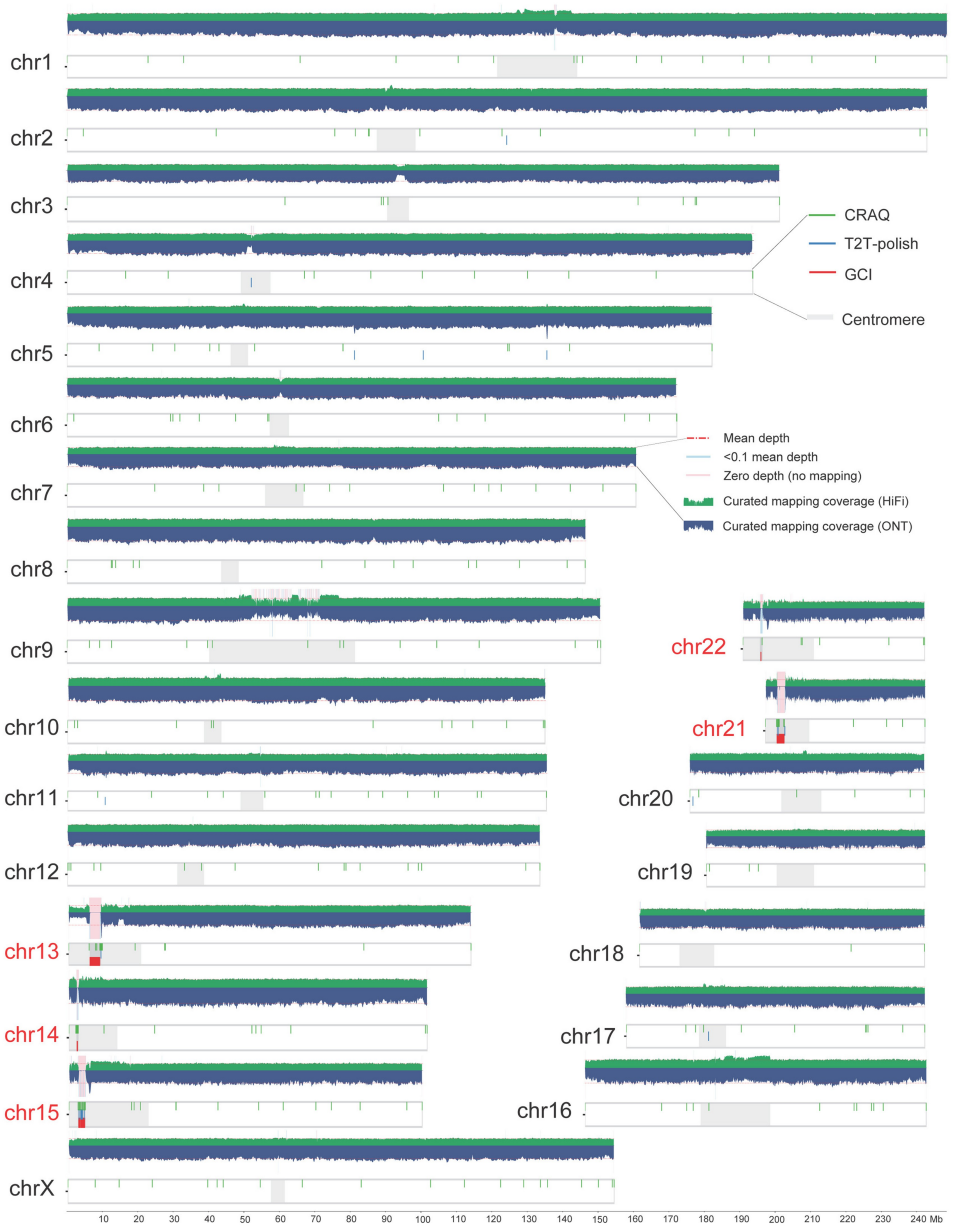
Assembly quality evaluation for human genome CHM13. Long read mapping and assembly issues as reported by GCI, T2T-polish, and CRAQ on CHM13 genome are shown. Horizontal dashed lines in the coverage plots represent the whole-genome mean mapping depth. Shaded regions suggest the regions with low high-confidence read mapping supports (<0.1 times the mean depth) and no support (zero depth), respectively. Five acrocentric chromosomes containing rDNA regions are highlighted.

Zooming in on the genomic regions of candidate issues in the CHM13 assembly, as detected by GCI, revealed that all 11 reported issues were located within the rDNA regions of five acrocentric chromosomes (Chr13, Chr14, Chr15, Chr21, and Chr22) (Fig. 3). Notably, these regions exhibited no high-confidence read mapping supports, with the longest issue spanning from approximately 6.01 to 8.78 Mb on Chr13. The rDNA regions are generally recognized as unresolved regions in all the currently available human genome assemblies, regardless of whether it is a haploid assembly or diploid phasing assembly (Nurk *et al.* 2022, Yang *et al.* 2023). Similarly, in the CN1 assembly, identified issues (76 and 63 for maternal and paternal) were significantly enriched in centromere regions (68 issues, *P* = 8.84e−90 for maternal; 58 issues, *P* = 2.52e−79 for paternal) (Supplementary Fig. S1), which suggested that these genomic regions required to be addressed further, potentially through more advanced sequencing technologies and improved assembling algorithms. In HG002, a total of 103 and 450 issue regions were identified for maternal and paternal haplotypes, respectively, including 37 and 364 centromeric issues (Supplementary Fig. S2).

### 3.3 GCI evaluation for genome assemblies of non-human model species

*Arabidopsis thaliana* and rice (*Oryza sativa*) are model species of dicot and monocot plants, respectively. For the *Arabidopsis* T2T assembly Col-CEN, its curated N50 value using HiFi reads (14.28 Mb) was significantly lower than that using ONT reads (25.74 Mb), which reached its theoretical maximum. The whole-genome GCI score for Col-CEN reached up to 100 when integrating both HiFi and ONT data (Table 1), likely due to its compact genome structure (135 Mb with only five chromosomes). Potential gaps were only detected near the telomeric region of chromosome 2 in the curated assembly, where a 45S rDNA region was located (Supplementary Fig. S3). Notably, extremely high coverage was observed in the region from ∼3.3 to ∼3.6 Mb on chromosome 2, due to the presence of a mitochondrial insertion, which was flagged as an issue by T2T-polish but not by GCI or CRAQ (Supplementary Fig. S3).

**Figure 4.**
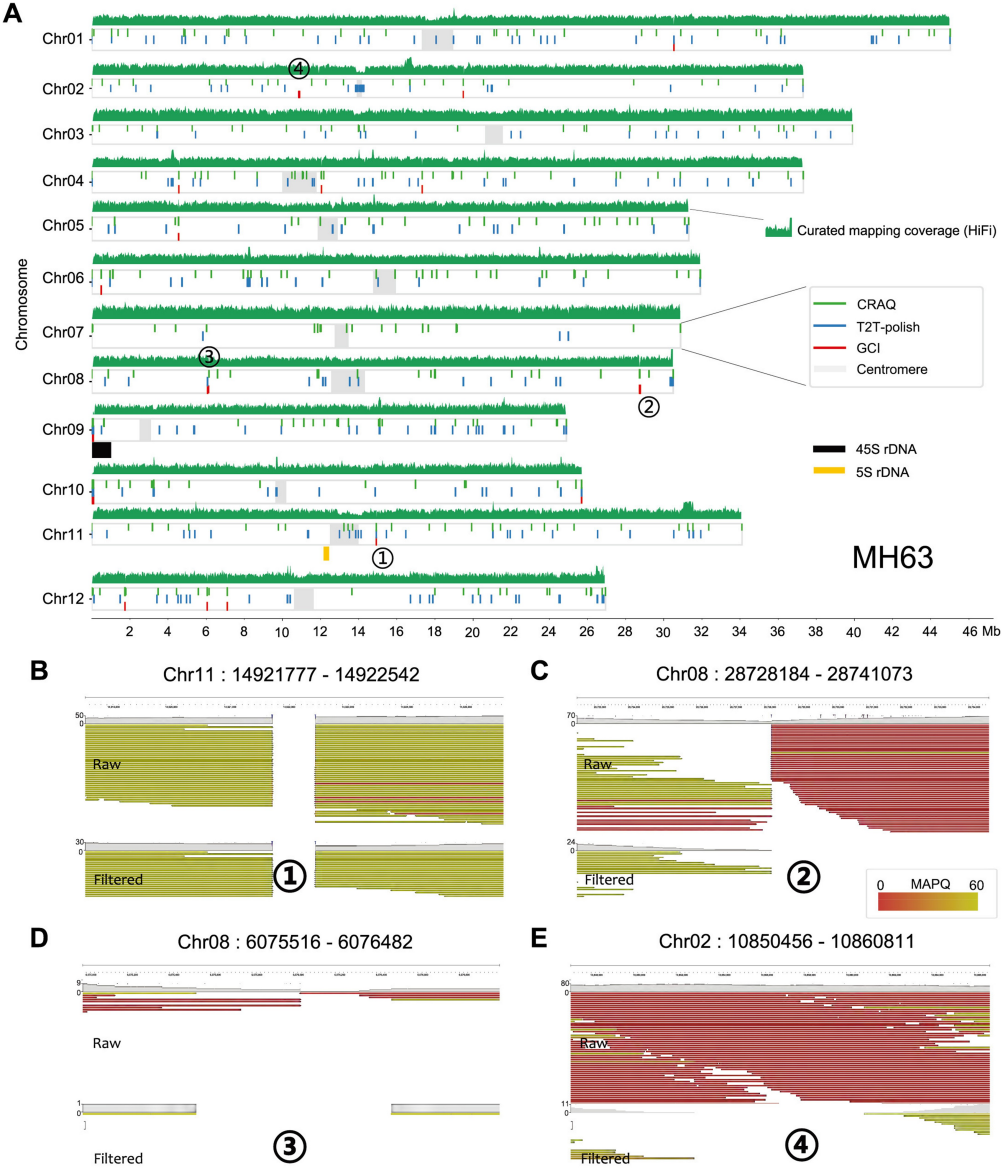
Assembly quality evaluation for rice genome MH63. (A) Genome-wide issues in the rice genome assembly MH63RS3 reported by GCI, T2T-polish, and CRAQ. HiFi mapping depth is plotted in sliding 1000 windows across each chromosome. (B) to (E) Genome browser screenshots highlighting four assembly issues in the MH63 (RS3) assembly. ONT reads alignments before and after filtration by GCI are shown.

Rice genome assembly MH63RS3 was the first gapless plant genome assembled using HiFi reads (Song *et al.* 2021). Evaluation using its HiFi reads yielded a GCI score of 49.89 (Table 1). The curated N50 value was 80% of its theoretical maximum, implying the presence of potential assembly issues. GCI detected a total of 21 issue loci, with problems observed in the 45S rDNA region on the distal end of chromosome Chr09, but not in the centromere or 5S rDNA regions (Fig. 4A).

Unlike the considerable number of so-called T2T genome assemblies reported in plants, only a few animal genomes have been reported to be completely assembled. For the recently released chicken complete genome assembly (GGswu), we assessed the quality with both HiFi and ONT data, yielding a GCI score of 29.37 and a curated N50 value of 73.41 Mb, which is 80% of its theoretical maximum (Table 1). Totally 582 issues across the genome were detected by GCI, spanning a total of 6.94-Mb regions, corresponding with the low GCI score (Supplementary Fig. S4). Specifically, 123 issues (1.12 Mb) were located on 10 macrochromosomes and 19 microchromosomes (totally 1046 Mb in length), primarily distributed in the telomeric regions of these chromosomes. Additionally, 434 issues (5.59 Mb) were detected on 10 dot chromosomes (40.7 Mb in total length), which implied that further validation is required to improve the assembly accuracy of these dot chromosomes.

### 3.4 Comparison with other tools

We compared the performance of CRAQ and T2T-polish with GCI in reporting assembly errors. Overall, GCI reported significantly fewer issues compared to CRAQ and T2T-polish (Table 2). This is largely due to the strategy of GCI in detecting assembly issues, which minimizes false positives by focusing on high-confidence read alignments and curated coverage, thereby avoiding the error introduced by non-assembly factors (e.g. sequencing and aligning bias). The CHM13 assembly is a well-recognized complete genome with few issues except for the rDNA regions, approved by limited issues reported by GCI (11/11 in rDNA regions) and T2T-polish pipeline (19/27 in rDNA regions). In comparison, CRAQ identified up to 328 issues, including 43 in rDNA regions (Fig. 3), many of which are likely false positives. In the case of the *Arabidopsis* genome, the 45S rDNA issues at the end of chromosome 2 were detected by all three tools. The mitochondrial insertion sequences (close to centromere of chromosome 2) were misidentified as an issue by T2T-polish, owing to its sensitivity to mapping coverage. For the chicken genome assembly, all tools reported numerous issues, reflecting its relatively low quality at the T2T level (Table 2; Supplementary Fig. S4).

**Table 2.** Numbers of assembly issues detected by GCI, CRAQ, and T2T-polish pipeline for model species genomes.

Species	Assembly (version)	GCI (no./length)	CRAQ (no.)	T2T-polish (no./length)
Human	CHM13 (v2)	11/5.99 Mb	328	27/0.29 Mb
Chicken	GGswu	582/6.94 Mb	1683	1829/44.67 Mb
*Arabidopsis*	Col-CEN (v1.2)	4/19.61 Kb	25	25/676.74 Kb
Rice	MH63 (RS3)	21/101.77 Kb	328	263/1733.06 Kb

For the rice genome assembly MH63 (RS3), 21 issues were detected by GCI, of which 15 overlapped with CRAQ and seven with T2T-polish issues (Table 2). The 45S rDNA region on the end of Chr09 showed issue signals from all three tools, while 5S rDNA region on Chr11 reported no issues by any tool (Fig. 4A). We manually examined the issue regions in the genome browser to verify assembly quality. Issue Chr11:14,921,777–14,922,542 was one of the five issues detected by all three tools (Fig. 4B). No high-confidence read alignment spanned this region and evident clipping signals were observed, suggesting a gap here. Issue Chr08:28,728,184–28,741,073 was identified by both GCI and CRAQ (Fig. 4C). Clipping information was captured by CRAQ to call this issue, and in the GCI workflow, removing clipped and low-quality alignments resulted in a gap here. Issue Chr08: 6,075,516–6,076,482 was shared by GCI and T2T-polish (Fig. 4D). Limited reads aligned to this region, which led T2T-polish to consider it as an issue. After filtration by GCI, no reads covered this region, therefore GCI also reported it as an issue. For GCI-specific issues, most were identified due to a lack of high-confidence read alignment support. For example, in issue region Chr02:10,850,456–10,860,811, no high-confidence reads spanned this area, leading GCI to report it as an issue (Fig. 4E).

Additionally, by detecting simulated issues in the *Arabidopsis* genome assembly Col-CEN, GCI outperformed the other two tools in precision, recall and F1 score overall (Table 3). Across the whole genome, GCI achieved comparable precision to T2T-polish and much higher than that of CRAQ. Summarized the results from multiple simulation runs, GCI demonstrated higher recall and F1 scores compared to CRAQ and T2T-polish. Despite the complex regions with highly repetitive sequences showed poorer performance compared to the simple regions across all the three tools, GCI proved to be more robust than both CRAQ and T2T-polish in these challenging regions.

**Table 3.** Performance of GCI, CRAQ, and T2T-polish in evaluating simulated assembly issues in the *Arabidopsis* Col-CEN genome.^a^

Run	Region	GCI			CRAQ			T2T-polish		
		Precision	Recall	F1 score	Precision	Recall	F1 score	Precision	Recall	F1 score
5 INSs + 5 DELs	Simple	**1.00**	**1.00**	**1.00**	0.50	0.66	0.57	**1.00**	0.82	0.90
	Complex	**1.00**	**0.50**	**0.67**	0.25	0.33	0.29	**1.00**	**0.50**	**0.67**
	All	**1.00**	**0.94**	**0.97**	0.47	0.62	0.53	**1.00**	0.78	0.88
10 INSs + 10 DELs	Simple	0.98	**0.98**	**0.98**	0.57	0.58	0.58	**1.00**	0.91	0.95
	Complex	**1.00**	0.92	0.96	0.30	0.25	0.27	**1.00**	**1.00**	**1.00**
	All	0.98	**0.97**	**0.97**	0.55	0.54	0.54	**1.00**	0.92	0.96
20 INSs + 20 DELs	Simple	**0.97**	**0.96**	**0.96**	0.63	0.69	0.66	0.96	0.83	0.89
	Complex	**1.00**	**0.50**	**0.67**	0.41	0.39	0.40	0.88	0.39	0.54
	All	**0.97**	**0.92**	**0.94**	0.61	0.66	0.63	0.95	0.79	0.86

a Best result for each run, region, and category is highlighted in bold.

## 4 Discussion

Producing a truly complete, contiguous, and accurate genome sequence is the ultimate goal of genome assembly efforts. The widespread application of long-read sequencing makes it feasible to obtain high-quality assemblies, including T2T assemblies. The commonly used quality metrics (e.g. N50/NG50/L50/auN, BUSCO/CEGMA, and QV) have proven insufficient for distinguishing nearly complete genome assemblies whose contig N50 values reach this species’ theoretical maximum (i.e. chromosome N50). Thus, an assembly quality inspector with a higher resolution is required to reveal potential assembly errors and detect gaps affecting the completeness of the assembly. Here we introduce GCI, a genome assembly quality evaluator at single-base resolution, to assess assembly continuity, by integrating long reads. Compared to CRAQ (Li *et al.* 2023), which collects clipping information of alignments to call assembly errors, GCI uses clipping information to filter alignments. Unlike the Flagger (Liao *et al.* 2023) and T2T-polish pipeline (Mc Cartney *et al.* 2022), GCI is not sensitive to read mapping coverage. In other words, CRAQ and T2T-polish pipeline call the assembly issues by processing abnormal or outlier clipping and depth signals, while GCI collects high-confidence continuous reads that support the correctness of local assembly. Although long-read sequencing is independent of PCR amplification and avoids GC bias, its sequencing bias can still be observed in complex repetitive regions. For example, HSat regions show coverage bias when HiFi (Pacbio Sequel II) and ONT reads are mapped, with mapping coverage decreasing to half of the whole-genome average depth for HiFi reads but doubling for ONT reads in the DYZ regions (Rhie *et al.* 2023). Therefore, long-read sequencing bias is a non-negligible factor that may introduce false positives of assembly issues detected based on read coverage. Additionally, the performance of long reads aligning in repetitive regions varies using different aligners (Jain *et al.* 2022) and improper use of aligning tools might cause mapping anomalies. Considering these factors, GCI incorporates alignments from multiple aligners and long-read sequencing platforms. Generally, GCI reports fewer issues than CRAQ and T2T-polish, yet provides more informative and precise coordinates for subsequent manual check. It should be noted that potential assembly issues reported by GCI, CRAQ, or T2T-polish may include misidentification, due to the variations in genome assembling and reads aligning algorithms to some extent. In other words, a correct assembly sometimes could be never approved by read mapping due to the highly repetitive sequence characteristics. For instance, high-confidence read alignment supports are usually not observed in 45S rDNA repeat sequences or telomere sequences, but this does not necessarily mean the assemblies are incorrect. Therefore, a sequence feature-aware assessment method is required to evaluate local assembly quality. The current version of GCI cannot capture issues arising from assembly collapse, while CRAQ and T2T-polish can identify such issues through clipping information or mapping depth. Therefore, these quality evaluators are complementary to each other to fully identify all categories of assembly issues, collectively contributing to the improvement of genome assembly quality.

## Supplementary Material

btae633_Supplementary_Data

## Data Availability

The GCI code is freely available on Github (https://github.com/yeeus/GCI) under the MIT open source license. Detailed issue regions for all assemblies evaluated in this study are available at https://github.com/yeeus/GCI/tree/main/benchmark. For the human genome CHM13, the raw reads and different versions of assemblies were obtained from Github (https://github.com/marbl/CHM13). All raw reads for HG002 were downloaded from https://github.com/human-pangenomics/HG002_Data_Freeze_v1.0, and the assemblies were downloaded from NCBI under the BioProjects PRJNA794175 and PRJNA794172. For CN1, the updated assembly version v0.9 was obtained from https://genome.zju.edu.cn. HiFi and ONT reads for the *Arabidopsis thaliana* genome Col-CEN (v1.2) were downloaded from ArrayExpress (accession E-MTAB-10272) and ENA (BioProject PRJEB46164), respectively. Illumina reads of the Col accession were downloaded from NGDC (CRA004538), and its assembly was obtained from Github (https://github.com/schatzlab/Col-CEN/tree/main/v1.2). For the rice genome MH63, the raw reads and assembly (RS3) were downloaded from NCBI (SRX6957825, SRX6908794, SRX6716809, and SRR13285939) and NGDC (BioProject PRJCA005549), respectively. The assembly and raw reads for the chicken genome GGswu are available in NCBI (BioProject accession PRJNA693184). Human genome assemblies from the HGSVC phase 3 were downloaded from The International Genome Sample Resource (https://ftp.1000genomes.ebi.ac.uk/vol1/ftp/data_collections/HGSVC3/).
