# Supplementary material for: GCI: a continuity inspector for complete genome assembly
Source: Bioinformatics. 2024 Oct 21;40(11):btae633. doi: 10.1093/bioinformatics/btae633 (PMC11550331; doi:10.1093/bioinformatics/btae633)
Supplement: btae633_Supplementary_Data [file btae633_supplementary_data.docx]

## Supplementary Information

**Supplementary Table S1:** Runtime and peak RAM usage of the GCI workflow in evaluating the assembly continuity of human, *Arabidopsis* and rice genomes.

| **Species** | Human | *Arabidopsis* | Rice |
| --- | --- | --- | --- |
| **Assembly** | CHM13v2 | Col-CEN | MH63 RS3 |
| **Assembly size (Mb)** | 3055 | 132 | 396 |
| **HiFi depth (×)** | ~58 | ~90 | ~39 |
| **MM2 paf (Gb)** | 3.6 | 0.32 | 0.44 |
| **WM2 bam (Gb)** | 115 | 9 | 11 |
| **ONT depth (×)** | ~134 | ~560 | NA |
| **ONT >100Kb depth (×)** | ~39 | ~4 | NA |
| **MM2* HiFi time / Peak RAM(t32**)** | 1.00h / 19.1Gb | 0.10h / 10.4Gb | 0.17h / 10.6Gb |
| **MM2 ONT time / Peak RAM(t32)** | 24.92h / 32.4Gb | 0.72h / 22.3Gb | NA |
| **WM2* HiFi time / Peak RAM(t32)** | 10.81h / 17.9Gb | 2.26h / 8.7Gb | 3.03h / 9.8Gb |
| **WM2 ONT time / Peak RAM(t32)** | 23.71h / 66.8Gb | 5.77h / 26.8Gb | NA |
| **GCI time / Peak RAM(t1**)** | 6.03h / 74.3Gb | 0.38h / 14.1Gb | 0.16h / 4.6Gb |
| **GCI time / Peak RAM(t32)** | 1.83h / 75.8Gb | 0.10h / 14.9Gb | 0.04h / 4.7Gb |
| **Total wall hours (t32)** | 62.27h | 8.95h | 3.24h |

* MM2, minimap2; WM2, winnowmap2; ** t32, 32 threads (-t 32); t1, single thread (-t 1).


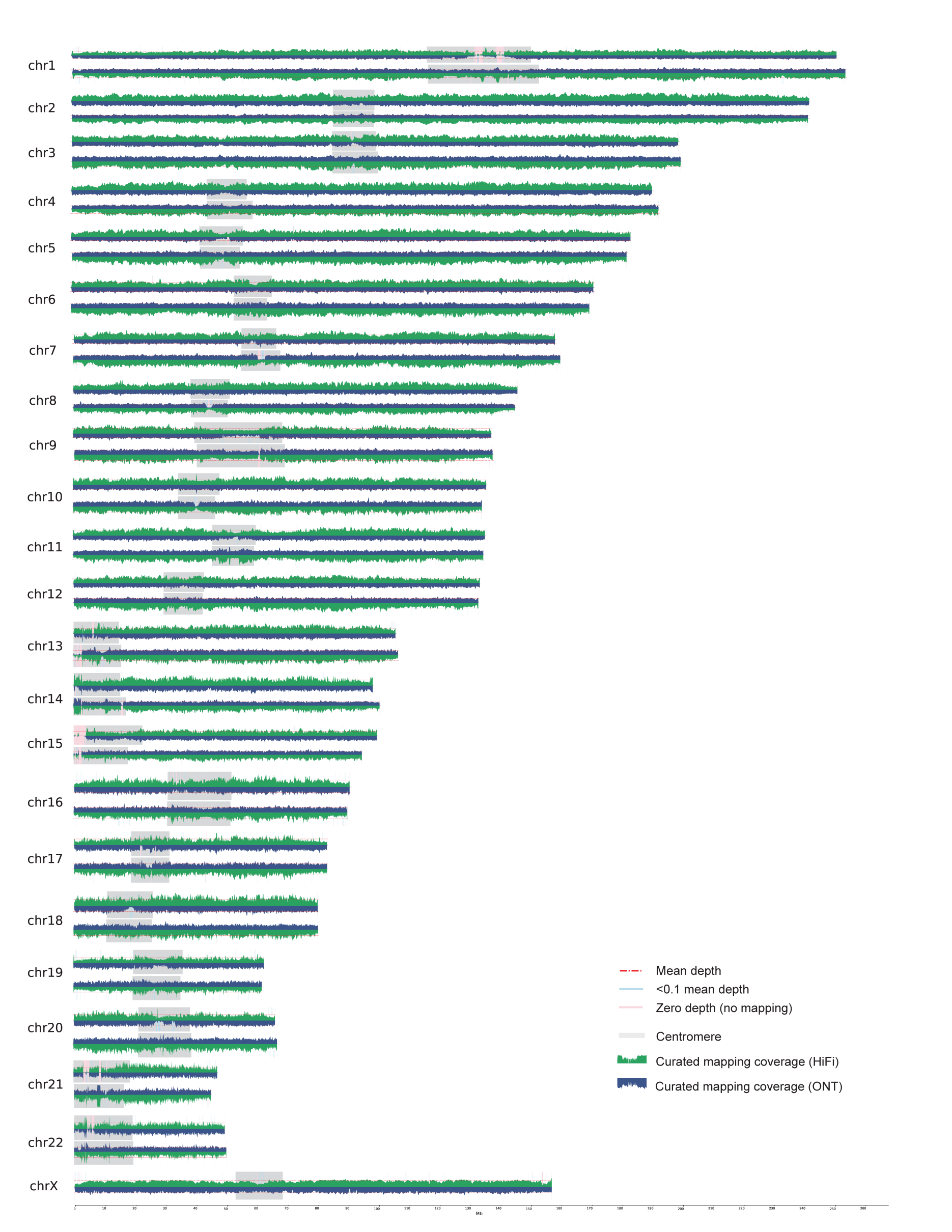


### Supplementary Fig. S1: Assembly quality evaluation for human genome CN1 using GCI.

Upper and lower tracks for one chromosome represent the mapping coverage of paternal and maternal assembly, where green and blue plots represent the coverage of HiFi and ONT read alignments. Horizontal red dashed lines indicate the whole-genome mean mapping depth. Light blue and pink shadows suggest the regions with low high-confidence read mapping supports (less than 0.1 times mean depth) and no support (zero depth), respectively.


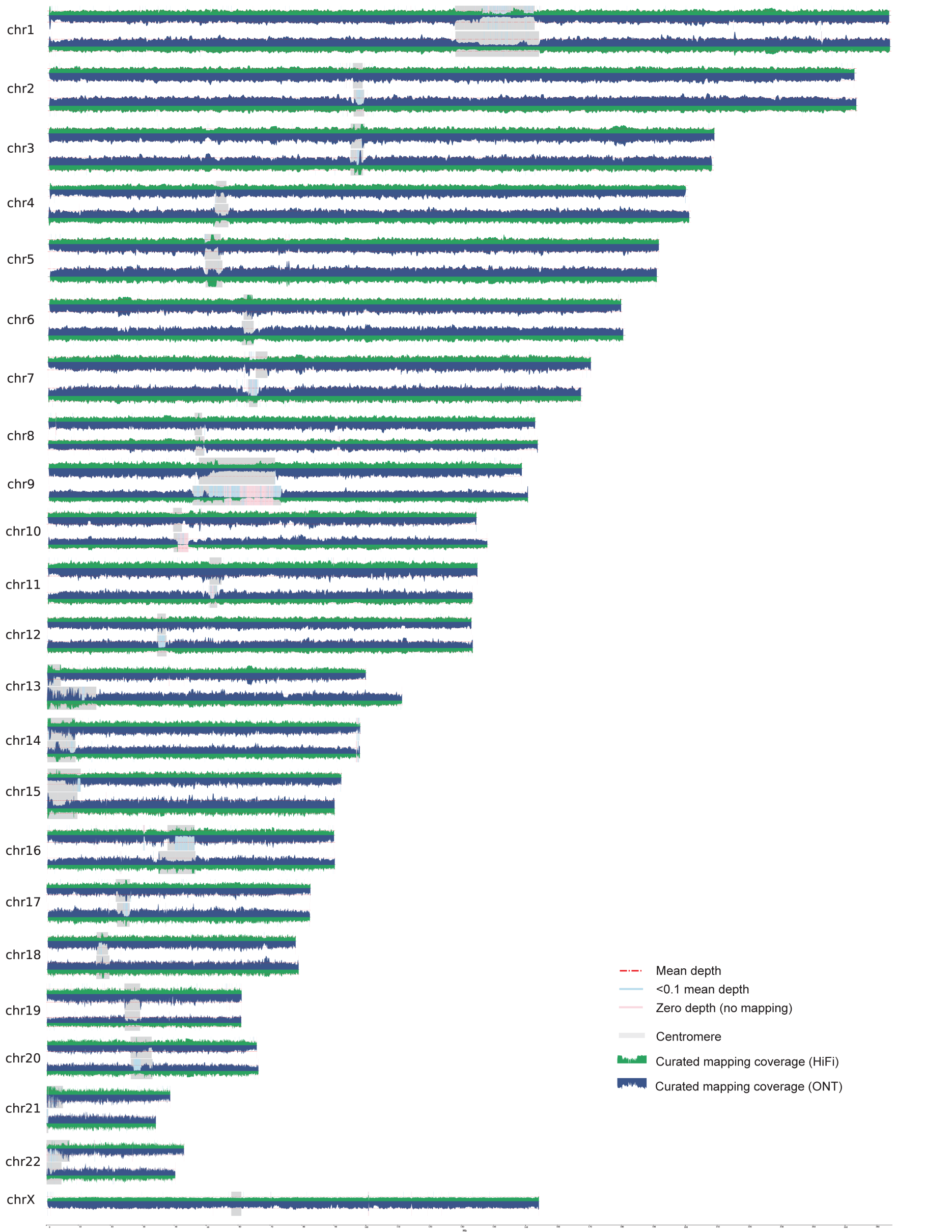


### Supplementary Fig. S2: Assembly quality evaluation for human genome HG002 using GCI.

Upper and lower tracks for one chromosome represent the mapping coverage of paternal and maternal assembly, respectively, where green and blue plots display the coverage of HiFi and ONT read alignments. Horizontal red dashed lines indicate the whole-genome mean mapping depth. Light blue and pink shadows suggest the regions with low high-confidence read mapping supports (less than 0.1 times the mean depth) and no support (zero depth), respectively.


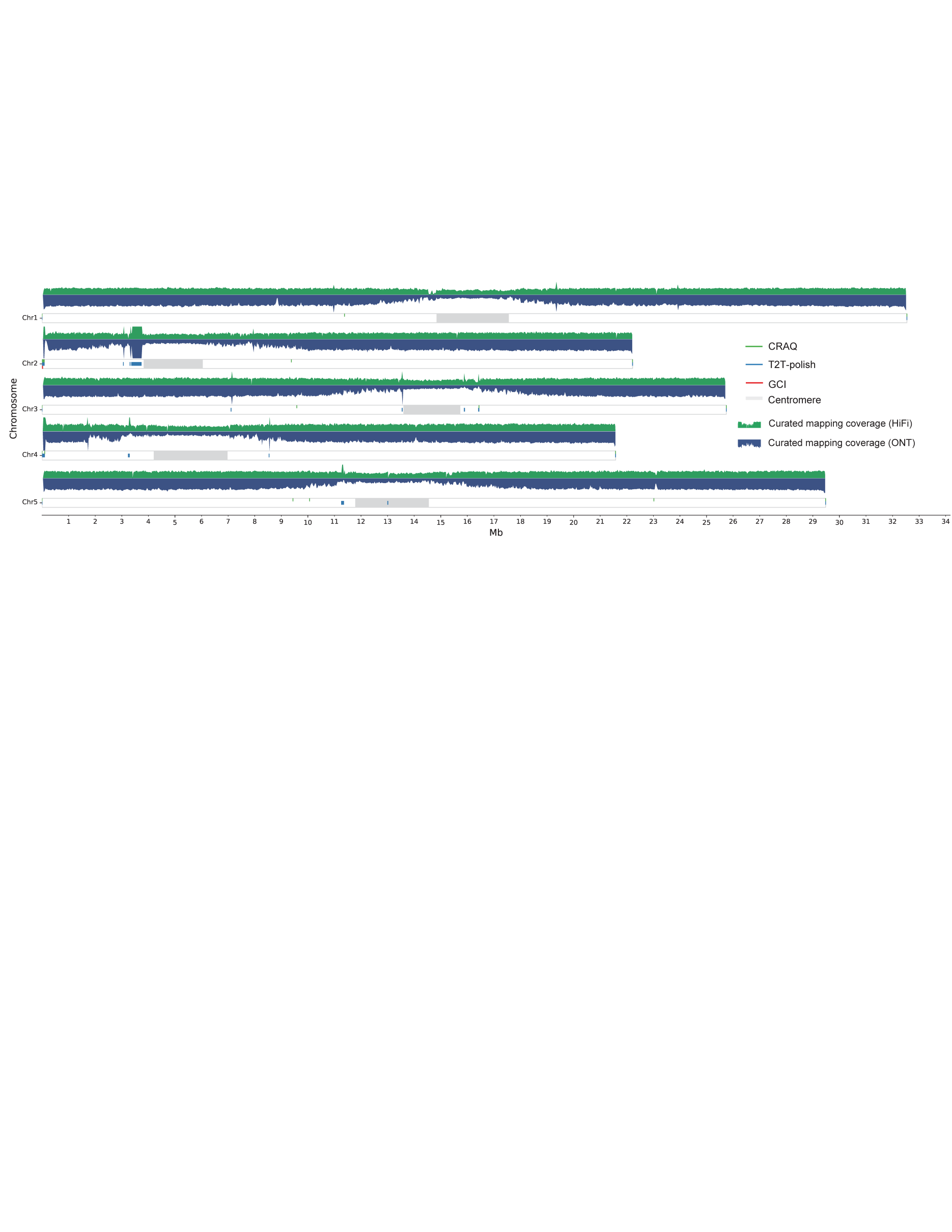


### Supplementary Fig. S3: Assembly quality evaluation for *Arabidopsis* genome Col-CEN using GCI, T2T-polish and CRAQ.


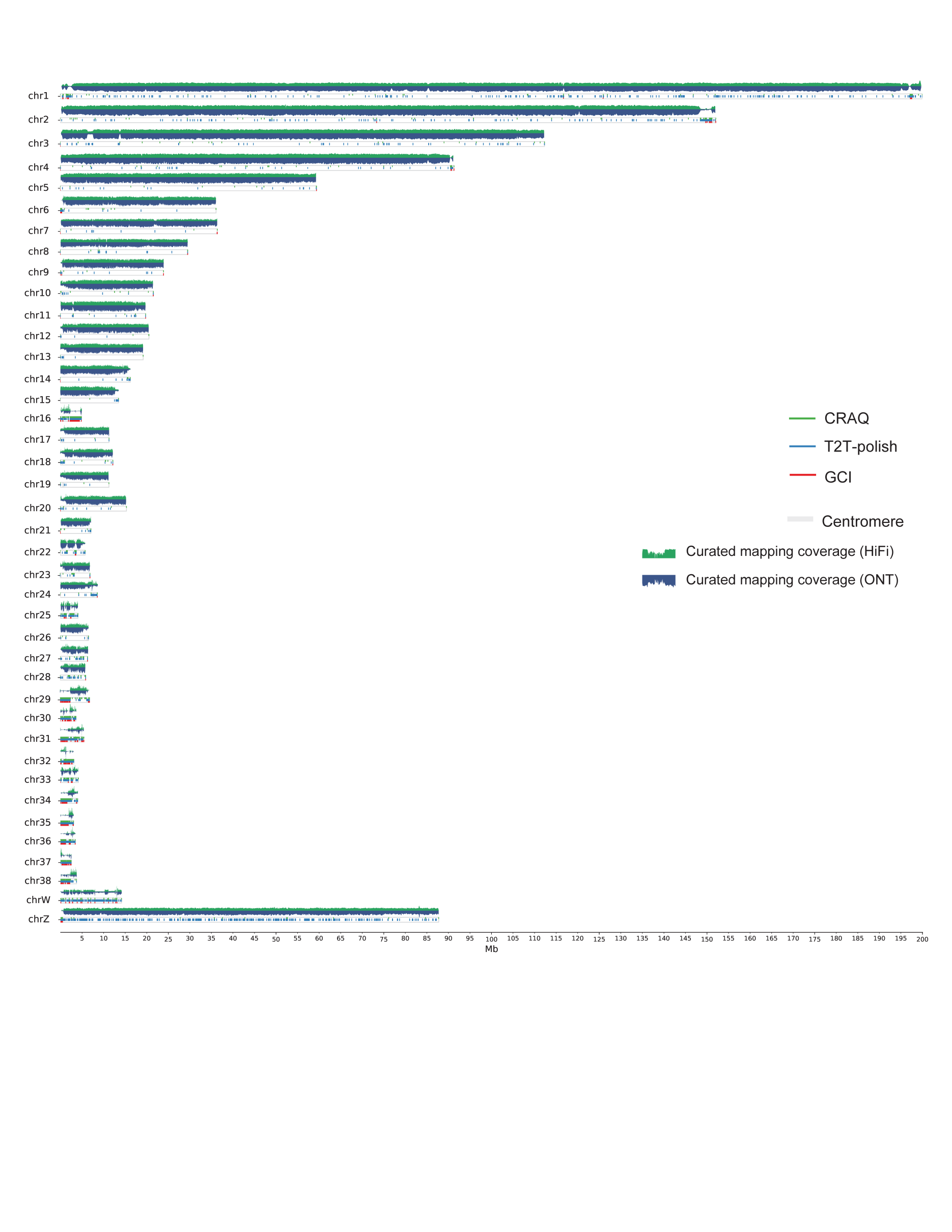


### Supplementary Fig. S4: Assembly quality evaluation for chicken genome GGswu using GCI, T2T-polish and CRAQ.
